# The impact of a vaccine scare on parental views, trust and information needs: a qualitative study in Sydney, Australia

**DOI:** 10.1186/s12889-017-4032-2

**Published:** 2017-01-23

**Authors:** Catherine King, Julie Leask

**Affiliations:** 1 National Centre for Immunisation Research and Surveillance, The Children’s Hospital at Westmead, Locked Bag 4001, 2145 Westmead, NSW Australia; 2 Discipline of Child and Adolescent Health, Sydney Medical School, The University of Sydney, The Children’s Hospital at Westmead Clinical School, Locked Bag 4001, 2145 Westmead, NSW Australia; 3 Sydney School of Public Health, The University of Sydney, Edward Ford Building A27, 2006 Westmead, NSW Australia

**Keywords:** Australia, Influenza, Immunisation, Vaccine, Safety, Parents, Attitudes, Communication

## Abstract

**Background:**

Vaccine safety scares can undermine public confidence in vaccines and decrease immunisation rates. Understanding and addressing parental concerns arising during such scares can assist in lessening their impact. In Australia in April 2010 there was a temporary suspension of influenza vaccine for children under 5 years of age after reports of an increase in the rate of adverse events following vaccination. This qualitative study aimed to explore the impact of the vaccine suspension on parental knowledge, attitudes, trust, information needs, and intent related to influenza vaccination and broader immunisation programs.

**Methods:**

Semi-structured interviews were conducted with 25 parents of children attending childcare centres in Sydney, Australia, between June 2010 and May 2011. Centres were selected to include parents from a range of socioeconomic backgrounds. Interview transcripts were coded and analysed using an approach informed by grounded theory.

**Results:**

Findings indicated that, for those who recalled the vaccine suspension, there was a lasting sense of uncertainty and confusion and a perceived lack of information. Parents had distinct information needs following the vaccine suspension, especially in regards to vaccine safety, testing and recommendations. For many, influenza vaccination intent was conditional on receipt of information from a trusted, authoritative source allaying safety concerns. Importantly, the impact of the scare was contained to influenza vaccines only, and not other vaccine programs.

**Conclusions:**

Parental concerns and information gaps following a vaccine safety scare need to be actively addressed. We provide policy and practice suggestions for proactively managing such incidents, particularly in relation to communication of timely, targeted information to parents and immunisation providers.

## Background

Vaccination safety scares have the potential to undermine public confidence in vaccines and lower immunisation rates, resulting in disease outbreaks and deaths. Scares may remain unsupported or eventually be supported by evidence. They may be amplified via mass media coverage and can have sustained impacts [1–3]. The United Kingdom’s (UK) measles-mumps-rubella vaccine (MMR) scare began in 1998 with an assertion that the vaccine could cause autism. This was refuted in further studies [4, 5], but still led to a reduction in MMR vaccine coverage in England from 91.8% prior to the scare to a low of 79.9% in 2004 [6] and resultant disease outbreaks [7]. The UK experienced a prior vaccine scare in the 1970s when the diphtheria-tetanus-pertussis (DTP) vaccine was associated with encephalopathy. Despite eventual evidence that this possible association was extremely rare [8], public confidence in the vaccine was eroded, including by negative media reportage [9], and immunisation rates dropped from 80 to 30% [10]. Subsequently, there was an outbreak of more than 300,000 pertussis cases, including 70 deaths [10]. In 1975 Japan suspended its whole-cell DTP vaccination program following the deaths of two children in December 1974 and January 1975 shortly after they received the DTP vaccine [11]. The program resumed in April 1975 [11] with a revised schedule delaying the first dose until 2 years of age [12]. Pertussis cases soared to over 13,000 in 1979, with 41 deaths [12] compared to pre-scare levels of 393 cases and no deaths in 1974 [11].

In April 2010 in Australia, an adverse event signal arose following an increase in notifications of febrile reactions, including convulsions, following influenza vaccination in children [13]. Following epidemiological investigations, there were 99 cases of febrile convulsions in children under 5 years of age determined to have a causal relationship to administration of seasonal influenza vaccine [14]. These convulsion cases occurred in states and territories across Australia, with the exception of the Northern Territory. A higher proportion (58 cases) were reported in Western Australia, where a state-based free influenza vaccination program for children aged 6 months to 5 years had commenced in 2008 [14].

Influenza vaccines have the potential to reduce influenza-like illness morbidity in children [15], reduce influenza virus transmission in the community [16] and provide some herd immunity benefit [17]. In Australia, influenza vaccination for young children above 6 months of age is recommended but only funded by the Australian National Immunisation Program (NIP), for those deemed susceptible to severe influenza disease or resultant complications [18, 19]. During the 2009 H1N1 influenza pandemic, a monovalent H1N1 vaccine was registered in Australia from December 2009 for children aged 6 months to 9 years and provided free of charge by the Australian Government [20]. The subsequent 2010 trivalent seasonal influenza vaccine included the H1N1 strain in combination with an A/H3N2 and a B strain but was only funded for those with identified risk factors [20].

Following detection of the adverse events signal related to influenza vaccine in children, the Chief Medical Officer of Australia suspended the use of all inactivated trivalent influenza vaccines for children under 5 years of age on 23 April 2010 [13]. An investigation was conducted into the possible cause (s) of the adverse events signal, and found that it was related to only one brand of influenza vaccine [21]. On 30 July 2010, use of other brands of influenza vaccines for children was resumed [22], while the suspension of the affected vaccine remained in force. An investigation into the affected vaccine, manufactured by bioCSL, continued and an official report was publicly released on 25 May 2011 [23, 24]. The eventual cause was theorised to be an interaction between viral components in the vaccine that resulted in an overly robust immune response and/or possible manufacturing process issues [25–27].

Parental confidence in vaccine programs can be a key factor influencing vaccine uptake [28]. Although vaccine safety scares and their impacts are well known and can include prolonged drops in vaccination rates, often years after the safety issue has been resolved [29], little research has investigated how parents themselves respond to these scares in their own words. Few if any have done so in real time during the evolution of the scare.

This study aimed to elicit an understanding of parental knowledge, attitudes and beliefs about the vaccine suspension. In particular, it aimed to explore the impact of the suspension on parental trust in influenza vaccine (including future influenza vaccination intent), emerging information needs and other immunisation programs.

## Methods

The study was conducted alongside a wider study examining the social, economic and health benefits of influenza prevention in children attending day care centres. This study was funded by an Australian Research Council grant. Ethics approval for the study was obtained from the Human Research Ethics Committee of The Children’s Hospital at Westmead.

One of the key research partners was a provider of early childhood education and long day care services in 72 centres across the Sydney metropolitan area. [30] The Australian Bureau of Statistics Socio-Economic Indexes for Areas (SEIFA) postcode data [31] was used in the selection of three centres for the study, to ensure inclusion of centres from different geographic and socioeconomic areas. The centres selected had SEIFA scores of 938.88, 1092.96, and 1122.7 respectively; a higher score is linked to a higher socioeconomic rating for that postcode area.

Parents with at least one child (aged 0–6 years) enrolled were recruited from the three selected childcare centres. Prior to recruitment, one researcher (CK) met with each childcare centre director to explain the study in detail. Information sheets about the project were provided to each centre director, who arranged for them to be displayed in the centre and emailed to all parents of children enrolled in the centre.

During recruitment, parents were generally approached as they were leaving the Centre after dropping off their child(ren); some parents directly approached the recruiter to ask about the study upon arrival at the Centre. Parent contact details were recorded and then parents were contacted by telephone or email (depending on parental preference) to organise an interview time. Care was taken not to oversample from any particular centre.

Semi-structured qualitative interviews (*n* = 25) were conducted by one researcher (CK). The interviews occurred over three key time-points during June/July 2010, October/November 2010 and May 2011, with different parents interviewed using the same interview schedule at each time-point. This allowed for the collection of data in the immediate aftermath of the vaccine suspension, after the resumption of the non-affected influenza vaccines for children, and prior to the beginning of the southern hemisphere 2011 influenza season, respectively. These time-points and major key events are noted in Table 1.

**Table 1:** Timeline of key events in relation to interview schedule

Date	Context	Interview timing
Mar 2010	Initial reports of febrile convulsions following receipt of seasonal influenza vaccine	
23 Apr 2010	Influenza vaccine suspended for children under 5 years of age	
		June 2010
2 July 2010	Therapeutic Goods Administration (regulator) initial report released	July 2010
30 July 2010	Use of non-bioCSL vaccines resumed	
24 Sept 2010	Therapeutic Goods Administration (regulator) updated report released	
8 Oct 2010	Therapeutic Goods Administration (regulator) overview report released	October 2010
		November 2010
25 May 2011	Final investigation findings released (day after last interview concluded)	May 2011

Participants were given the option of face-to-face or telephone interviews at a time and location convenient to them. All participants elected to participate in telephone interviews. Consent for study participation and recording of the interviews was obtained from each participant.

Topic domains covered in the interview included parental knowledge and thoughts about influenza disease (both seasonal and pandemic H1N1), influenza vaccines for children and the vaccine suspension, including its impact on trust and intent for both influenza vaccination and other vaccination programs. Parental information needs, including preferred information formats and delivery mechanisms, were also explored in relation to the vaccine suspension.

Throughout the interviews, the interviewer (CK) used intra-interview checking techniques to ensure that the participant responses had been correctly understood. Notes were taken during the interviews concurrent with audio recording. Participants were encouraged to consult a medical practitioner for answers to any specific clinical questions about their child(ren) arising during interviews.

Interview recordings were transcribed word-for-word by a professional transcription service shortly after a batch of two to three interviews was conducted. This timely transcription allowed for ongoing analysis by the researchers to guide the subsequent interviews. Interviews were conducted until theoretical saturation of concepts was reached with no additional divergent cases identified.

Transcripts were imported into qualitative research software NVIVO 10 (QSR International, 2012). Line-by-line coding was undertaken and coding was initially linked to major question areas. The authors met to discuss and refine emerging higher order themes. Analysis was informed by grounded theory methodology, which emphasises the derivation of theory from the data [32]. Particular attention was paid in the analysis to seeking out exceptions diverging from the predominant findings.

Information on the key time-points of major events associated with the vaccine suspension was collected (see Table 1). This, together with situational analysis which stems from grounded theory and maps key influences existing throughout the research period [33], was used to explicitly consider the complex context that existed at the time of the study to aid understanding of the participants’ responses. This included the recent 2009 influenza pandemic (which although milder than early predictions still caused an estimated 123,000–203,000 deaths worldwide in 2009) [34], development and use of a monovalent pandemic vaccine and subsequent use of a trivalent vaccine including the pandemic strain, and finally the suspension of the vaccine. Broader societal factors and potential influences on parents and the linkages between these were also mapped to inform the analysis.

## Results

Twenty-five semi-structured interviews were conducted. Of these, 9 interviews were conducted in June/July 2010, 8 in October/November 2010 and 8 in May 2011. A summary of participant demographic information is contained in Table 2.

**Table 2:** Demographic information – interview participants

Parents	24 mothers, 1 father
Age	under 21 years (*n* = 1) 21–30 years (*n* = 2) 31–40 years (*n* = 19) 41–50 years (*n* = 3)
Country of birth	Australia (*n* = 17) Overseas (*n* = 8)
Education	University degree (*n* = 16) TAFE (vocational training college) (*n* = 2) Completed 6 years secondary school (*n* = 5) Completed 4 years secondary school (*n* = 2)
Employment	Outside the home (*n* = 18) Home duties (*n* = 7)

Situational analysis of the context for the interviews identified the following key constructs with the potential to intersect with the emerging themes from the interviews: societal concepts of disease; disease experiences – both personal and of contacts; proximity to perceived risk; parental time constraints; influencers – family, friends and health professionals; and broader societal parenting expectations.

Pseudonyms are used in the reporting of the findings to preserve the confidentiality of interview subjects, with direct quotes also listing the month and year obtained to enable transparency as to the contextual background. The following themes emerged from the analysis and were evident and consistent across the three interview timepoints in the study: ‘Uncertainty and unease’, ‘Interaction with existing perspectives’, ‘Tried and true – the ‘normal’vaccines’, ‘Mind the gap’ - the information void’ and ‘Bridging the gap – parental information preferences’.

### Uncertainty and unease

The majority of interviewees had some knowledge that a vaccine suspension had occurred. However, this knowledge was largely incomplete and characterised by confusion as to what had happened, where it had occurred and what vaccines were involved. Some participants recognised that influenza vaccine was involved, but were unsure whether it was the H1N1 ‘swine flu’ vaccine or the seasonal influenza vaccine.

The overall recollection of parents was a negative one created by the overriding perception that something ‘bad’ had happened with children perhaps killed or severely adversely affected. As outlined by Renuka in July 2010, three months after the vaccine suspension occurred,

I have information stored in my brain that it’s gone wrong and after that there was no news at all.

This negative impression persisted even in the interviews completed after the non-bioCSL vaccines were available again. As Marina interviewed in May 2011, thirteen months after the suspension relayed,

I don’t know if kids died, or something was wrong with the vaccine, and they just stopped giving or something like that.

Most of the parents interviewed had heard directly about the vaccine suspension from the media including via television reports, radio, online sources and newspapers. No parents mentioned knowing that other influenza vaccines for children were available for use, despite 16 of the 25 interviews being conducted after use of the non-affected vaccines had resumed.

A sense of unease about the influenza vaccine for children was evident from parental responses. Those who had previously contemplated vaccinating their child(ren) against influenza were now hesitant to do so. For example, as explained by Penny in July 2010,

I was debating on whether to do it and then there were those cases on TV where the people had kids who’d got sick …so I left it.

This unease was related in part for some parents to the reponsibilty they felt as parents making health decisions for their child(ren). As articulated by Tara in June 2010,

it is a bit alarming you know thinking that you know moving forward if you were to get the injection you know, would some of this happen? The kids are obviously so precious as you know, and you would be to blame, like me as the parent. I mean yes, there would obviously be someone else to blame, but you would feel '…you knew about this, you had it done and then you know something's happened'.

Interestingly, some parents indicated that they would proceed with their own influenza vaccinations, despite a reluctance to immunise their child(ren) against influenza. As outlined by Rachel in October 2010,

I have no intention of taking them [the children] in to get the flu shots next year, unless something triggers me to do it, whereas just for me, yes I will go and get my flu shot.

### Interaction with existing perspectives

Parents’ pre-existing beliefs about influenza disease and the influenza vaccine were an important context in which they interpreted the vaccine suspension. Many parents believed influenza to be a very mild illness, which in part appeared attributable to a general confusion about what constituted influenza disease compared to the common cold. In describing both her own confusion and that of others Yasmin stated in October 2010,

I get very confused about colds and flus and things like that, and I suppose a general cold isn’t that serious, but a flu can sometimes be a little bit more debilitating …I think most people refer to whatever they’ve got, whether it be a cold or anything else, it’s always referred to generally as a flu.

Actual experience of influenza illness lent clarity to the distinction between colds and influenza, with influenza then being perceived as very serious. For some parents, the H1N1 influenza pandemic had heightened awareness and concerns about influenza.

For others the pandemic and its attendant media attention had functioned to de-escalate concerns about influenza, in particular when initial predictions about pandemic severity were not borne out in people’s experience.

The high level of dread associated with other illnesses was not apparent for many parents, allowing them to rationalise concerns and establish a view of influenza vaccine as unnecessary. As mentioned by Jessica in October 2010,

If a child gets polio or rubella, I think the consequences can be life threatening…but my understanding is generally if you treat flu well, and your children don’t have any underlying health problems, it’s not usually a fatal illness … in my mind the other illnesses are much more serious.

Many parents felt their child(ren) were healthy enough to resist influenza infection and/or complications and that it could be prevented by handwashing and a healthy diet. Some parents were confused by the need for annual vaccination, with variable awareness of the need to match the vaccine to currently circulating influenza strains. Others thought it may be beneficial to allow their child to contract influenza to build immunity from the wild disease, rather than by vaccination.

Parents who had existing concerns about influenza vaccination for their child(ren) solidified their opposition to it following the vaccine suspension. Several parents stopped or delayed plans to have their child(ren) vaccinated against influenza. For some the decision was also related to practical barriers such as lack of time.

For one respondent the vaccine suspension provided reassurance that Australia has a robust vaccine safety system. For this parent, the fact that a problem vaccine was identified, removed and investigated by relevant authorities was evidence that the system was working effectively.

### Tried and true – the ‘normal’ vaccines

The impact of the suspension on parental trust appeared to be limited to the influenza vaccine alone. For most participants, the suspension had no impact on their confidence in other vaccines or established vaccination programs. Parents expressed familiarity with vaccinations they had received as a child, with the success and longevity of these programs contributing to a sense that these vaccines were well known, understood and adequately tested.

The perceived stability of the formulation of existing vaccines was contrasted with the ever-changing nature of influenza vaccines. Parents were concerned that the annual need to change formulations may leave inadequate time for proper testing. As discussed by Rachel in October 2010,

Other vaccinations yes, they’re proved and tested vaccines, whereas …the flu ones, because they keep changing yes I think they’re the riskier ones.

The established funded NIP was seen as an essential and routine part of childhood by virtually all the parents interviewed. In contrast, the exclusion of influenza vaccine from the funded program suggested to some parents that it was at best optional, and at worst potentially unsafe. As articulated by Ingrid in July 2010,

I don't have a problem getting them immunised from anything else – probably because it's been a government issued, um, almost stipulation now and it's paid for by the government, so I suppose I maybe naively think that they must be completely safe …. whereas the flu one is still voluntary, and I think well if it's voluntary maybe it's not safe enough.

A divergent view was expressed by one parent, Mandy, interviewed in November 2010, who felt that the vaccine suspension had impacted negatively upon her view of all vaccines, making it difficult for her to allow her child to have any vaccines. This hesitancy was linked to other vaccine scares (MMR) with her stating “the whole autism thing rings in my ears”.

### ‘Mind the gap’ - the information void

In contrast to long-established vaccine programs, many parents described a lack of information about the influenza vaccine suspension. Initial reporting about the suspension but then no further updates left parents unsure what to do, and allowed for the persistence of a negative impression about influenza vaccination of children. As expressed by Alexandra in May 2011,

I don’t have any up-to-date information, so I don’t know what's happened since then, what they found. I remember that there was some controversy and they suspended it, but …especially now it’s coming up to winter, I don’t know what the updated situation is on that, or whether it’s okay to do the vaccine or not.

Conflicting advice from their General Practitioner (GP) added to confusion for some parents with some GPs still advising that no influenza vaccines were recommended for children under 5 years of age as late as May 2011, despite non-affected vaccines being officially resumed for children less than 5 years of age from 30 July 2010.

Clear information gaps emerged throughout the interviews. Some parents expressed a desire to know more about the severity of influenza disease, the need for, and composition of, influenza vaccine for children and how seasonal strain change affects vaccine testing. Many parents advanced specific and detailed information needs about the suspension, particularly concerning safety, testing and investigative outcomes. For example, as Anna queried in May 2011,

what the likelihood of side-effects are and who it’s been recommended by and what kind of cover it provides?

Despite the regulatory authority, the Therapeutic Goods Administration conducting investigations and writing reports throughout the period of the interviews (see Table 1), no parent reported hearing about the existence or outcomes of these. Question areas about the suspension articulated by parents included:

- What exactly occurred?
- Who was affected?
- Why were they affected?
- What side effects were there?
- What is in the vaccine?
- What were the existing recommendations?
- What are the recommendations post-suspension?
- Is it safe now?
- What testing has been done?
- What was the outcome of an investigation?
- Who has recommended the use of this vaccine?

The provision of information from a reliable source to meet these information needs was a pre-condition for most parents to contemplate influenza vaccination of their child(ren). As stated by Ingrid in July 2010,

So until a report comes out, either on the TV or the newspaper, that tells us that there've been some successful studies now and it's all okay, uh, until then, I wouldn't consider it.

A small number of parents would still consider vaccinating their child(ren) against influenza. Reasons advanced for this included the future commencement of school by their child(ren), living with family members vulnerable to severe influenza infection, the impending birth of a baby, and potential financial difficulties if the primary income earner contracted influenza.

### Bridging the gap – parental information preferences

Parents mentioned a range of information sources, formats and delivery mechanisms that would be useful for meeting their expressed information needs. Great importance was placed on information being from a trustworthy and reputable source.

GPs were acknowledged as a trusted source of information for many. However, obtaining information from GPs was not always seen as a practical solution as GPs were not immediately accessible and only consulted if a family member was unwell.

Instead, freely accessible, authoritative sources of information were preferred.

As Elena stated in June 2010,

I wouldn't go out of my way to go visit a GP just to ask these questions – but by all means if it was online on some sort of you know, secure government website, then yes, absolutely.

Parents mentioned a range of formats and distribution mechanisms useful for suspension-related information. This included broadcasts via traditional media sources such as radio and television, as well as hard copies of information in brochure or poster form at locations including childcare centres, healthcare centres and libraries. There was recurrent mention of lack of time availability impact on when and how information could be accessed. For many parents this led to a strong preference for internet-based information, which could be accessed after hours.

Unfortunately, for some parents there was now a gap in trust levels that would be hard to overcome with information provision alone. As stated by Paula in May 2011,

I mean it probably wouldn’t matter what was said now, I would feel that there was also a chance that something could go wrong.

## Discussion

This study provides detailed, contextualised information about how parents of young children responded to a vaccine safety scare. To our knowledge this is the only qualitative study examining parents’ responses to the Australian 2010 influenza vaccine scare at key time points as it evolved. The scare did not arrive in a vacuum of perceptions and beliefs about the influenza vaccine. Rather, it interacted with existing views whereby parents used the narrative in diverse ways to make meaning about child health, risk, influenza and its vaccine. The study also found that parents experienced considerable uncertainty about the vaccine that stemmed from a gap in information provision in the post-suspension period and that this uncertainty was evident at each interview timepoint. Their knowledge of the vaccine suspension was minimal, with many having a negative recollection of events with very little precision in their knowledge of the suspension and resumption of non-affected vaccines. Having identified this gap, many openly expressed a desire to know more, including via the mass media and other passive sources, although only a few expressed an intention to actively seek out information.

The vaccine suspension, affecting as it did young children, could be considered an event of high ‘dread’ [35] in which there is the potential for a high level of what Sandman terms ‘outrage’ [36]. The negative gist parents associated with the influenza vaccine can be explained by fuzzy trace theory. This asserts that the gist response to an event can often be more strongly recalled than the detail of the event itself and risk information processed according to the essence of the recollection [37, 38]. Individual meanings, pre-existing knowledge and feelings interact to try to achieve sense-making about an issue [39]. In this study, initial hesitancy about influenza vaccine being viewed as optional, less necessary and constantly changing was amplified by the suspension and functioned to validate parental concerns.

Parental decision making about vaccination risk, is complex and individualized [40, 41]. The situational analysis conducted as part of this study illuminated the many complex structural and social systems parents interact with and that form a backdrop to risk processing. Parents, especially mothers, are subject to increased scrutiny about health decision making for their child(ren) from both peers and wider society [42]. In parallel to this, increasingly sophisticated technologies and bodies of knowledge make it impossible for individuals to completely understand all aspects of every topic, forcing some reliance on experts to translate these to a lay audience [43, 44].

In this study, advice from ‘experts’, in this instance GPs, about the safety of the vaccine sometimes conflicted with regulatory advice. Another Australian study noted that practitioner confusion existed around the post-suspension recommendations, with 13% of GPs advising against receipt of the childhood influenza vaccine during the period 2010–2012 [45].

Negative media reporting can lessen uptake of vaccination during and/or following vaccine safety scares [46, 47]. Mass media reportage of issues is driven by what is considered currently newsworthy [35, 48]. During the vaccine suspension, government agencies reacted to determine the cause of the higher rate of adverse events, a process that necessarily took considerable time. Media attention moved on from the suspension story. The absence of clear messages from trusted heath authorities via the media at this time created an information void for parents, inadvertently allowing the perpetuation and persistence of a negative association. Parents did not understand the nuance around one vaccine remaining suspended while the other vaccines were deemed safe, and vaccination intent was affected.

A qualitative study conducted in the UK to examine parents’ views of the MMR vaccine controversy determined that health scares increase parental information needs, particularly in relation to future vaccination intent [49]. Recent evidence suggests that vaccine hesitant parents may not necessarily be persuaded by information provision and well-reasoned argument [50]. However, it appears that for many study parents, in the circumstance of the suspension, vaccination was conditional on the provision of a clear safety message via authoritative sources. A government report [24] was finalised but not publicly released before the completion of this study, so it is not known what impact, if any, this would have had on parents’ views.

The qualitative findings in this study, that confidence in the NIP vaccines largely remained while confidence in influenza vaccines was eroded, are supported by quantitative Australian studies, including a survey conducted in 2010 among parents whose child had experienced an adverse event from a vaccine (including both influenza and NIP vaccines) [51]. Another survey, conducted in Western Australia where a funded program existed, examined the impact of the paediatric influenza vaccine suspension on vaccination coverage rates. It found that childhood influenza vaccination rates decreased from 45.5% in 2009 to 7.9% in 2010 and 17.3% in 2011, but there was no decrease in coverage rates for the NIP-funded vaccines [52]. A further study tracking vaccine uptake and parental attitudes towards the influenza vaccine for young children from 2010–2014, concluded that vaccine uptake remains low following the 2010 adverse events, with 41.7% of children fully vaccinated in 2008–2009 compared to 7.1% in 2010–2014. This decrease was noted both in healthy children and in those with comorbidities placing them at a higher risk of influenza disease. There was a slight rise in influenza vaccine uptake in children with comorbidities in 2014, however, the results were not statistically significant [53].

### Limitations

This study contained a majority of highly educated parents, thus may have under-represented the views of less educated parents, despite sampling from childcare centres across a diverse range of socioeconomic areas.

### Implications for public health

There is a need for public health authorities to be proactive during an adverse events incident and engage with both parents and providers. We have shown that agencies cannot rely on the message getting through via other sources or parents actively seeking out information. Well-established crisis communication principles should be utilised. These include keeping the many publics (and professionals) informed, acknowledging uncertainty and showing respect for concerns expressed [35, 36, 54, 55]. Thus, even in the absence of clear findings from an investigation, some attempts to keep the public informed should be undertaken, including communication of what is known and not yet known and what is being done to address the gaps.

Information should be tailored to different audiences to meet them at their point of need, with a balance of straightforward messages backed by further details for those who require this [35]. In the instance of the suspension, parents requested clear, consistent follow-up advice and recommendations for action particularly when approaching key decision points such as the beginning of the new influenza season. Trust needs to be rebuilt in regards to the safety of the influenza vaccine and specific information gaps met, including via two-way dialogue [35] with parents.

Given the fragmented sources accessed by parents, information should be disseminated via multiple means. Traditional media sources should be used, but given the likelihood that media interest may quickly wane, other trusted sources should also be utilised, such as distributing information resources via childcare centres and providing authoritative information on government health websites. Although not mentioned in this study, the proliferation and increase in use of social media sites since the study make it likely that dissemination using this means may also be beneficial.

Immunisation providers, such as GPs and nurses, should be specifically targeted with up-to-date information, including ready-made resources to discuss with or give to parents. Finally, capacity in health systems should be built to ensure the ability to cope with increased information needs during and after health scares, including detailed planning for who will communicate during a vaccine safety scare and how.

## Conclusion

This qualitative study found that the suspension of the influenza vaccine for children under 5 years of age had a negative impact on parental views about that vaccine during June 2010-May 2011. Parents in this study displayed hesitancy about their children being vaccinated against influenza. For most parents, their concern was linked to perceived inadequate information provision. The negative impact of future vaccine scares could potentially be mitigated by the provision of clear, concise information about the progress of investigations, the research and testing undertaken, and by giving clear direction about when vaccination can be resumed.

Reassuringly, even though this event generated uncertainty around the influenza vaccine, with the exception of one divergent case it did not affect trust for long-established vaccination programs. However, as demonstrated by the impact on immunisation rates from other vaccine scares, public health officials need to be proactive in dealing with future vaccine scares and ready to disseminate clear, targeted information to both parents and immunisation providers.
